# Role of pilocarpine use following laser peripheral iridotomy in eyes with refractory acute angle closure glaucoma: A case report and literature review

**DOI:** 10.1097/MD.0000000000029245

**Published:** 2022-07-08

**Authors:** Chu-Yu Yen, Chun-Chen Chen, Po-Chen Tseng

**Affiliations:** a Department of Ophthalmology, Taipei City Hospital, Renai Branch, Taipei, Taiwan; b Institute of Clinical Medicine, National Yang-Ming University, Taipei, Taiwan.; c Department of Ophthalmology, School of Medicine, College of Medicine, Taipei Medical University, Taipei, Taiwan.; d Department of Special Education, University of Taipei, Taipei, Taiwan.

**Keywords:** angle closure glaucoma, cataract surgery, intraocular pressure, laser peripheral iridotomy, pilocarpine

## Abstract

**Rationale:**

Angle closure glaucoma (ACG) is one of the most emergent types of glaucoma in clinical practice. Laser peripheral iridotomy (LPI) could minimize pupillary block and prevent ACG from an acute attack. However, recurrent increase in intraocular pressure (IOP) may still occur despite successful LPI. The aim of this study is to highlight the importance of postLPI pilocarpine use and larger LPI size as well as to share some experiences of cataract surgery in patients with ACG.

**Patient concerns:**

A 63-year-old female was referred to our hospital for headache, and poor control of IOP in the right eye for 3 hours.

**Diagnoses:**

The patient was diagnosed ACG in the right eye. Recurrence of ACG in the right eye and new-onset and recurrent ACG in the left eye were noted during follow-up, despite successful LPI. The diagnosis was confirmed through slit lamp and gonioscope examination.

**Interventions:**

The LPI size was enlarged and pilocarpine use was maintained at 2% (1 drop 4 times a day) in both the eyes. Finally, cataract surgery was performed in both the eyes.

**Outcomes:**

No recurrence of ACG was noted during postLPI pilocarpine use in both the eyes. The postoperative IOP was stable for >6 months after cataract surgery without any surgical intervention or antiglaucoma medication use. No discomfort or major complication was observed.

**Conclusion:**

This report highlights the importance of postLPI pilocarpine use and larger LPI size in patients with refractory ACG.

**What Was Known/What This Paper Adds**

**What Was Known**

- ACG, one of the most emergent types of glaucoma, can be initially managed with LPI to control IOP before cataract surgery.
- However, the appositional closure from an anteriorly positioned ciliary body or the development of peripheral anterior synechiae may cause aqueous humor blockage and result in ACG recurrence.

**What This Paper Adds**

- PostLPI pilocarpine use and enlargement of LPI size before cataract surgery may decrease the recurrence rate and yield better IOP control in patients with recurrent ACG.
- The outcome of this method is favorable in both the eyes in our case. The IOP could be maintained in an ideal range in clinical practice.

## 1. Introduction

Globally, cataract and glaucoma are the leading causes of visual loss. It is estimated that 67.9% and 7.1% of all blindness cases in individuals aged ≥50 years are attributed to cataract and glaucoma, respectively.^[1]^ Angle closure glaucoma (ACG), which is attributed to outflow obstruction of aqueous humor, is one of the most emergent types of glaucoma. The mechanism responsible for angle closure mainly involves in the pupillary block, in which the contact between the iris and the lens at the pupillary margin impedes the flow of the aqueous humor into the anterior chamber, eventually causing aqueous blockage at the pupillary margin and trabecular meshwork.^[2]^ The association of cataract and ACG has been widely discussed. In the advanced stage of cataract-mediated pupillary block, the thickened lens and consequent angle closure lead to intraocular pressure (IOP) elevation.^[3]^ Traditionally, the management of ACG involves laser peripheral iridotomy (LPI) to alleviate pupillary block and prevent the development of an acute attack.^[2]^ However, an increase in IOP still occurs in 46.7% patients during the 18 month follow-up despite successful LPI.^[4]^ For these patients, cataract surgery to remove the thickened lens can increase anterior chamber depth and prevent subsequent IOP rise.^[5,6]^ Despite the successful experiences of treating ACG using LPI and cataract surgery, few studies have mentioned about preventing the recurrence of ACG during LPI and cataract surgery. In this paper, authors highlight the importance of larger LPI size and postLPI pilocarpine use as well as share few experiences of cataract surgery in patients with ACG.

## 2. Case report

A 63-year-old female without systemic diseases presented with headache, fullness, and poor control of IOP for 3 hours in the right eye despite being prescribed topical Cosopt (2% dorzolamide + 0.5% timolol) (1 drop thrice a day), brimonidine 0.15% (1 drop thrice a day), and travoprost 0.004% (1 drop at bedtime) at the local clinic. Her ophthalmologic history revealed an episode of acute angle closure glaucoma (AACG) in the right eye, for which she was administered intravenous (IV) mannitol (200 mg/mL, 500 mL) immediately and underwent LPI at 10 o’clock and 12 o’clock positions on August 11, 2021. A subsequent measurement revealed an IOP of 10 mm Hg, and the patient has been receiving antiglaucoma medications since then.

**Figure 1.**
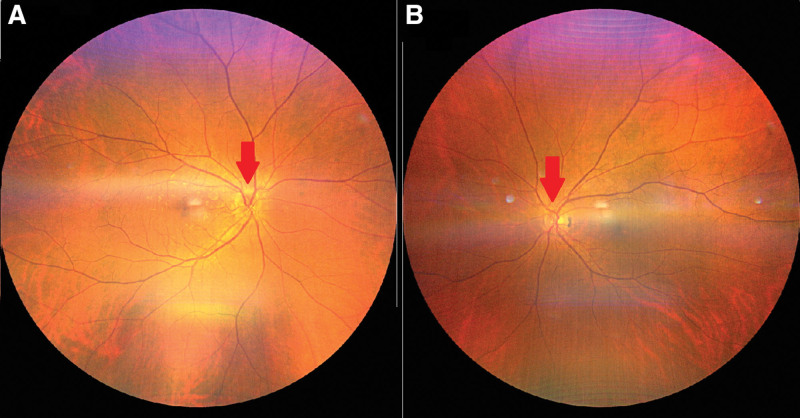
Fundus of the patient. Note the crowded disc in both the eyes (arrows). A: right eye; B: left eye.

**Figure 2.**
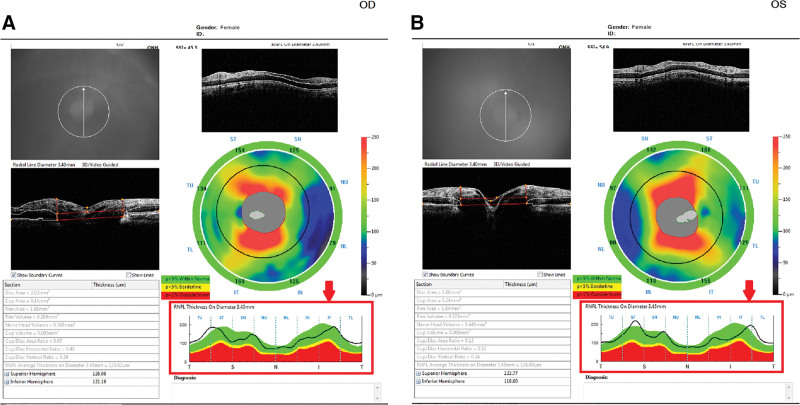
Optical coherence tomography. The thickness of the retinal nerve fiber layer was within the normal range in both the eyes (arrows). A: right eye; B: left eye.

The patient visited our hospital on August 18, 2020, owing to visual discomfort in the right eye. The results of a physical examination are shown in Table 1. The IOP in the eye was 41 mm Hg. The best corrected visual acuity (BCVA) according to the Snellen chart was 0.3. In addition, diffuse microcystic corneal edema was observed. Gonioscopy without indentation revealed 360º invisible posterior trabecular meshwork. Moreover, anterior chamber inflammation with 4+ cells was noted. Iris scars were noted at 10 and 12 o’clock positions in the eye. LPI was performed to puncture a size of approximately 50 µm at the 12 o’clock site. The pupil was found to be mid-dilated with poor light reflex. In addition, severe nuclear sclerosis with pupillary block was noted. Subsequent fundus photography and optical coherence tomography demonstrated crowed disc and normal retinal nerve fiber thickness (Figs. 1, 2).

**Table 1:** Results of physical examination of the patient.

	Right	Left
IOP (mm Hg)	41	9
BCVA	0.3 (bare)	0.6 (+ 2 D sph/-3 D cyl axis 25°)
Axial length (mm)	2.47	2.10
Cornea	Diffuse microcystic edema	Clear
Angle	a.360º of invisible posterior trabecular meshwork	a.Visible posterior trabecular meshwork
	b.Schaffer grading: 0	b.Schaffer grading: 2
Anterior chamber	Shallow, cells 4+	Shallow, clear
Iris	a.Scar at 10 o’clock and 12 o’clock sites	Normal appearance
	b.LPI: 12 o’clock site	
	c.Iris bombe	
	d.Mid-dilated pupil with poor light reflex	
Lens	Severe nuclear sclerosis	Severe nuclear sclerosis
Vitreous	Clear	Clear
Retina and optic nerve	Crowded disc	Crowded disc

Abbreviations: BCVA = best corrected visual acuity, IOP = intraocular pressure, LPI = laser peripheral iridotomy.

**Figure 3.**
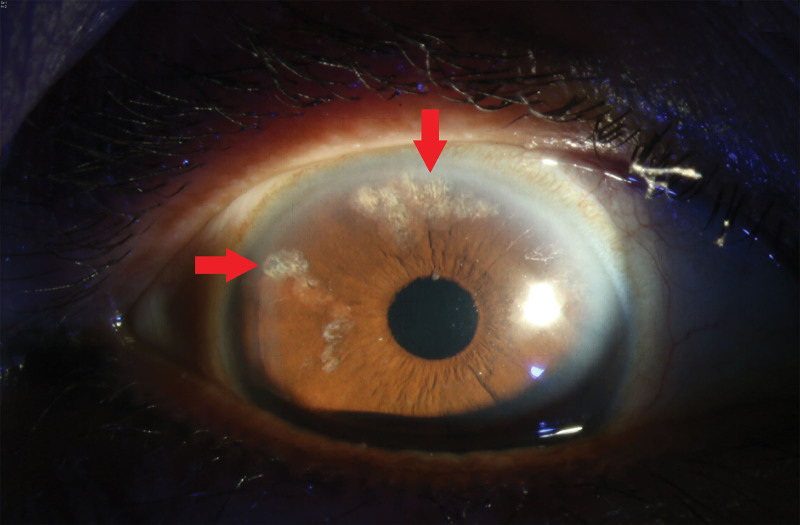
Six months after cataract surgery in the right eye. Note the LPI scars at 10 o’clock and 12 o’clock sites (arrows).

Under the impression of recurrent AACG, 1 bottle of IV mannitol (200 mg/mL, 500 mL) was immediately administered. In addition to antiglaucoma medications, pilocarpine 2% (1 drop 4 times a day) and prednisolone 1% (1 drop 4 times a day) were administered to prevent LPI occlusion and to decrease inflammation, respectively. The LPI size was increased to 200 µm at the 12 o’clock site the next day. Follow-up IOP was <12 mm Hg for over >1 month. Subsequently, cataract surgery was scheduled on October 6, 2021. The patient consent was obtained and was informed regarding the general and severe complications of ocular surgery, including endophthalmitis. During the surgery, the lens cortex was found to be firmly adherent to the capsule bag; thus, we carefully performed hydrodelination first and then aspirated the residual material with irrigation and aspiration cannula. postoperatively, the patient was stable. Follow-up IOP at 6 months was 8 mm Hg, with a BCVA of 1.0 and deepened anterior chamber (Fig. 3).

Another episode of AACG in the left eye occurred on October 20, 2021. The presentation was similar to that in the right eye. The IOP was 43 mm Hg with a shallow anterior chamber. Gonioscope showed pupillary block and 360º invisible posterior trabecular meshwork indentation. Considering AACG, 1 bottle of IV mannitol (200 mg/mL, 500 mL) was administered immediately, in addition to Cosopt (2% dorzolamide + 0.5% timolol; 1 drop thrice a day), brimonidine 0.15% (1 drop thrice a day), travoprost 0.004% (1 drop at bedtime), pilocarpine 2% (1 drop 4 times a day), and prednisolone 1% (1 drop 4 times a day). The IOP was 10 mm Hg 1 day after the treatment. Subsequently, LPI was performed to ensure a size of 200 µm at the 12 o’clock site. On October 27, 2021, the IOP was 10 mm Hg and LPI was patent.

**Figure 4.**
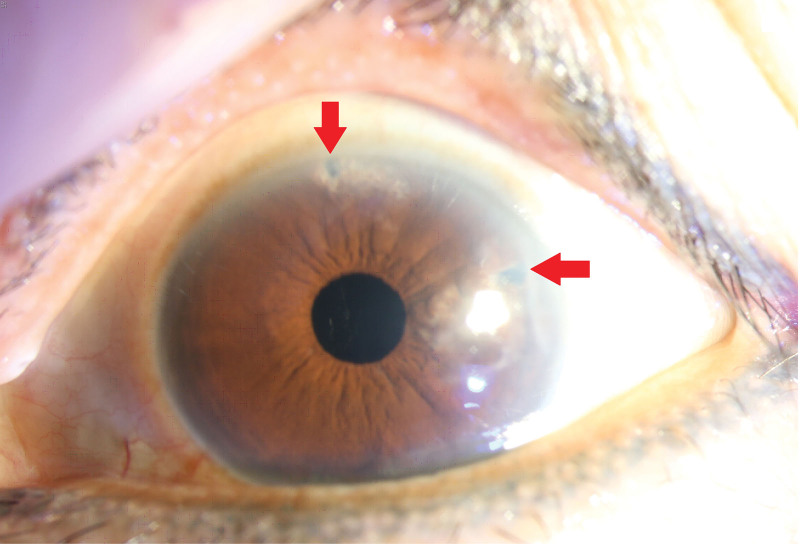
Six months after cataract surgery in the left eye. Note the LPI scars at 2 o’clock and 12 o’clock sites (arrows).

However, AACG recurrence was noted in the left eye on November 09, 2021. The IOP was 41 mm Hg, and the LPI was found to be occluded. A review of medications used revealed that the patient had discontinued pilocarpine use since November 08, 2021, because the eye drops were missing. Since midnight at that day, she experienced pain and fullness in the left eye because of IOP elevation. She was prescribed IV mannitol (200 mg/mL, 500 mL) immediately and was prescribed pilocarpine 2% (1 drop 4 times a day), while maintaining antiglaucoma drug use and enlarging LPI to 300 µm at 2 and 12 o’clock sites. Fortunately, the AACG did not recur thereafter, and the IOP was 12 mm Hg on November 23, 2021. We then conducted cataract surgery on December 01, 2021. During the surgery, the iris prolapsed frequently. After phacoemulsification, 3 mL of carbachol 0.01% was injected into the anterior chamber, and no iris prolapse occurred after the operation. Follow-up IOP was 12 mm Hg at 6 months, with BCVA of 1.0 and deepened anterior chamber (Fig. 4).

## 3. Discussion

The key point of refractory ACG lies in the pupillary block caused by the thickened lens, which occupies almost all the potential space of the aqueous humor outflow pathway. Thus, although successful LPI could yield satisfactory outcomes in the first few months, further cataract surgeries are often unavoidable.^[4–6]^ The EAGLE study conducted in United Kingdom found that even in patients without cataract, clear-lens extraction resulted in lower mean IOP, requiring lower glaucoma medications, lesser additional glaucoma procedures, and better visual acuity than LPI.^[5]^ Lam et al further found that cataract surgery could decrease the cumulative incidence rate of IOP rise to only 3.3% at 18 months.^[4]^ These findings imply that early cataract surgery can be considered as a definite and first-line treatment to prevent IOP rise in patients with ACG.

Considering the limitation of LPI, we attempted our best to perform cataract surgery as soon as possible in both the eyes after ensuring successful LPI, IOP stabilization, and decreased inflammation in the anterior chamber. Unfortunately, in both the eyes, AACG recurred before cataract surgery despite initial successful LPI. The reason underlying the recurrence in the eyes were found to be different. In the right eye, the cause was the smaller LPI size initially. Fleck suggested that the LPI should be at least 150 to 200 µm in diameter if acute angle closure glaucoma is to be reliably prevented.^[7]^ In clinical practice, however, the LPI is often difficult to conduct at the initial presentation owing to severe corneal edema and increased iris thickness during an AACG attack. In such cases, prednisolone 1% and pilocarpine 2%–4% are useful to decrease corneal edema, reduce iris thickness, and facilitate perforation.^[8,9]^ The initial LPI performed at the local clinic was of only 50-µm size and posed a higher risk for recurrence. This experience highlighted the importance of LPI size. If a larger LPI size cannot be urgently ensured, frequent visits to the outpatient department and early enlargement of the LPI are necessary.

Second, this case highlighted the importance of pilocarpine use despite successful LPI. In the left eye, 200 µm of LPI was adequate; however, AACG recurrence was noted only one day after the cessation of pilocarpine use. Reportedly, pilocarpine is effective in pulling peripheral iris away from the trabecular meshwork to open the anterior chamber angle.^[10]^ It is well-known that pilocarpine can constrict pupil, make the iris thinner, and help in subsequent LPI.^[11,12]^ Despite the initial successes of LPI, 38%–58% patients subsequently had persistently raised IOP.^[13,14]^ This might be attributed to the appositional closure from an anteriorly positioned ciliary body or the development of peripheral anterior synechiae.^[15]^ To the best of our knowledge, no study has reported about the role of pilocarpine use after successful LPI. We suggest that despite successful LPI, pilocarpine cessation may still decrease the size of LPI and causes pupillary block in the scenario of pupil dilation, such as at midnight or while using mydriatic agents.

During cataract surgery, few considerations may be required in patients with ACG. Posterior capsular rupture, retrobulbar hemorrhage, and zonular dehiscence are the mostly encountered aspects.^[16]^ In our case, the lens cortex was firmly adherent to the capsule bag, allowing easy posterior capsular tear. This highlighted that in patients with ACG who underwent cataract surgery, careful hydrodelination is necessary. It not only prevents the risk of capsular tear but is also easy to perform for practitioners who do not have an expertise in glaucoma management. In our patient, iris prolapse was noted in the left eye during cataract surgery, indicating that the iris was not stable as in floppy-iris syndrome. Previous studies have suggested that the use of atropine 1% drops 3 times a day, starting 1 to 3 days before the surgery, and intraoperative intracameral injection of epinephrine 0.2 mL (1:100 000 epinephrine) help make incision location more central; this is followed by intracamerol injection of 3 mL carbachol 0.01% into the anterior chamber after phacoemulsification. These adjustments during cataract surgery may stabilize the anterior segment structure and make the operation easier and safer.^[17]^

Our study still features several limitations. First, although we noted that the outcomes of patients with ACG who underwent patent LPI were highly associated with pilocarpine use, the exact mechanism remains unknown. Second, although the final IOP and visual acuity after cataract surgery were fine in both the eyes, there exists a slim chance for ACG recurrence. In addition, the mechanism other than pupillary block could not be delineated. However, the concept of postLPI pilocarpine use, larger LPI size, and surgical tips in patients with ACG may improve surgical outcomes to some extent. Further studies with a longer follow-up and a larger sample size are still needed to confirm these results.

## 4. Conclusions

LPI is indicated to treat ACG. However, some patients still experience recurrent ACG despite successful LPI. This report highlights the importance of postLPI pilocarpine use and larger LPI size in patients who have refractory ACG.

## Author Contributions

Conceptualization, P.C.T. and C.Y.Y.; methodology, P.C.T. and C.Y.Y.; validation, P.C.T. and C.Y.Y.; resources, P.C.T. and C.Y.Y.; writing—original draft preparation, C.Y.Y.; writing—review and editing, P.C.T.; visualization, P.C.T.; supervision, P.C.T., C.C.C. All authors have read and agreed to the published version of the manuscript.
